# Supplementary figures and images for: Risk-sensitive foraging does not explain condition-dependent choices in settling reef fish larvae
Source: PeerJ. 2020 Jan 13;8:e8333. doi: 10.7717/peerj.8333 (PMC6964687; doi:10.7717/peerj.8333)

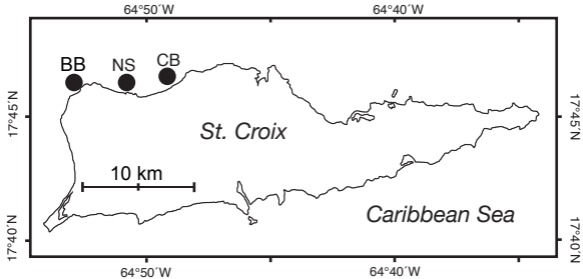

Supplement: Figure S1 — Sites: BB = Butler Bay, NS = Northstar, CB = Cane Bay [file peerj-08-8333-s001.pdf]

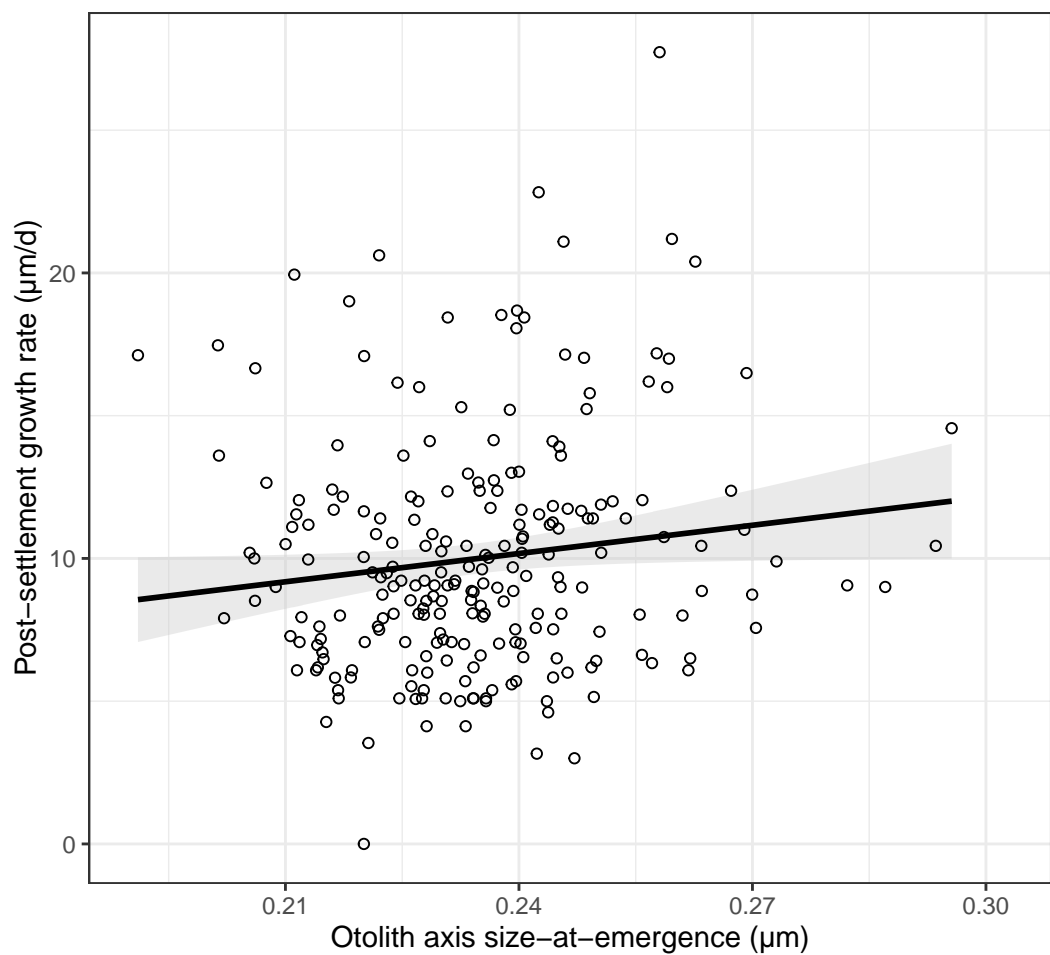

Supplement: Figure S2 — Each point represents one fish. The curve is a linear regression with 95% confidence interval (R2 = 0.01). [file peerj-08-8333-s002.pdf]

Post-settlement age

8  
6  
4  
2  
0

Solitary

Group

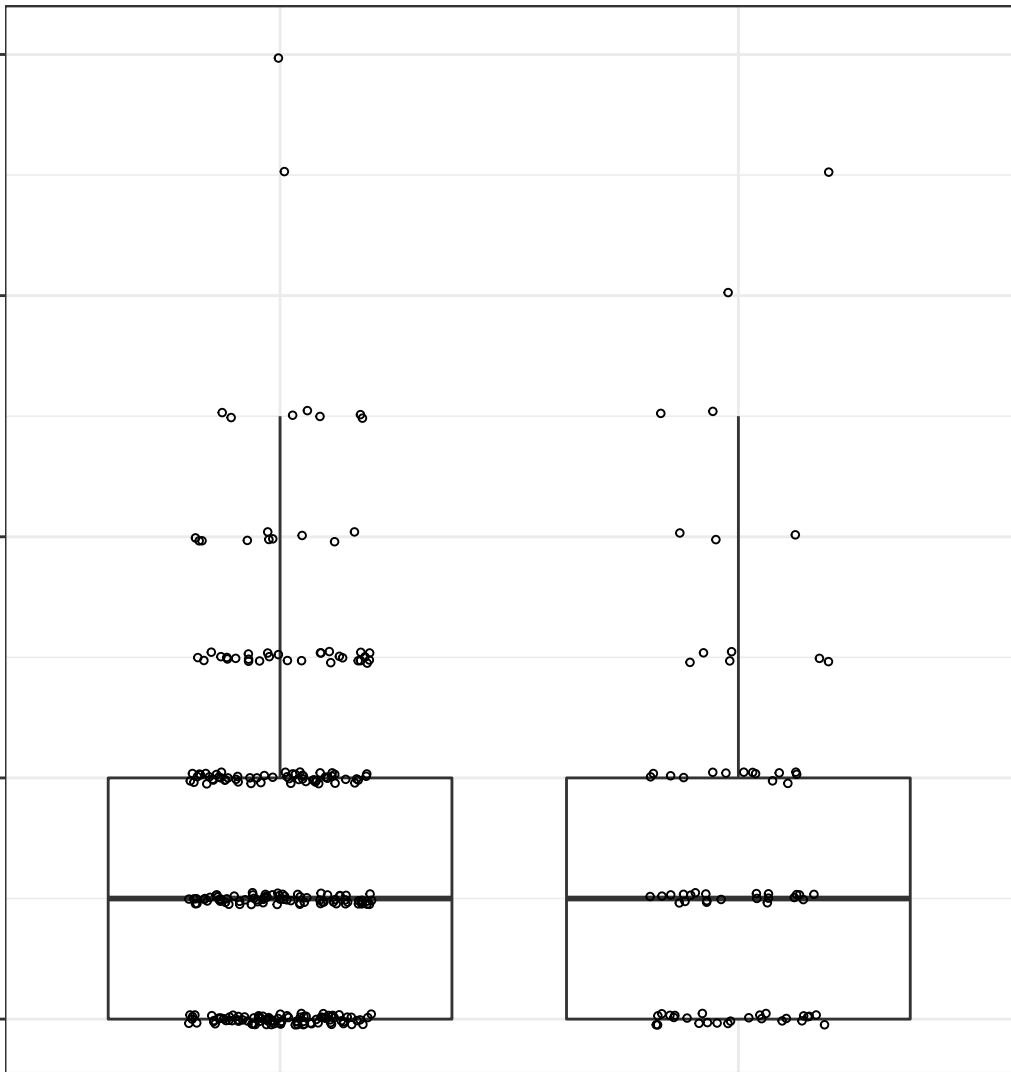

Supplement: Figure S3 — Age distributions of the solitary and grouped fish used in the analysis. Boxplots show median, interquartile range, and the 95% quantile range, along with individual data points. Data were jittered for visibility. [file peerj-08-8333-s003.pdf]

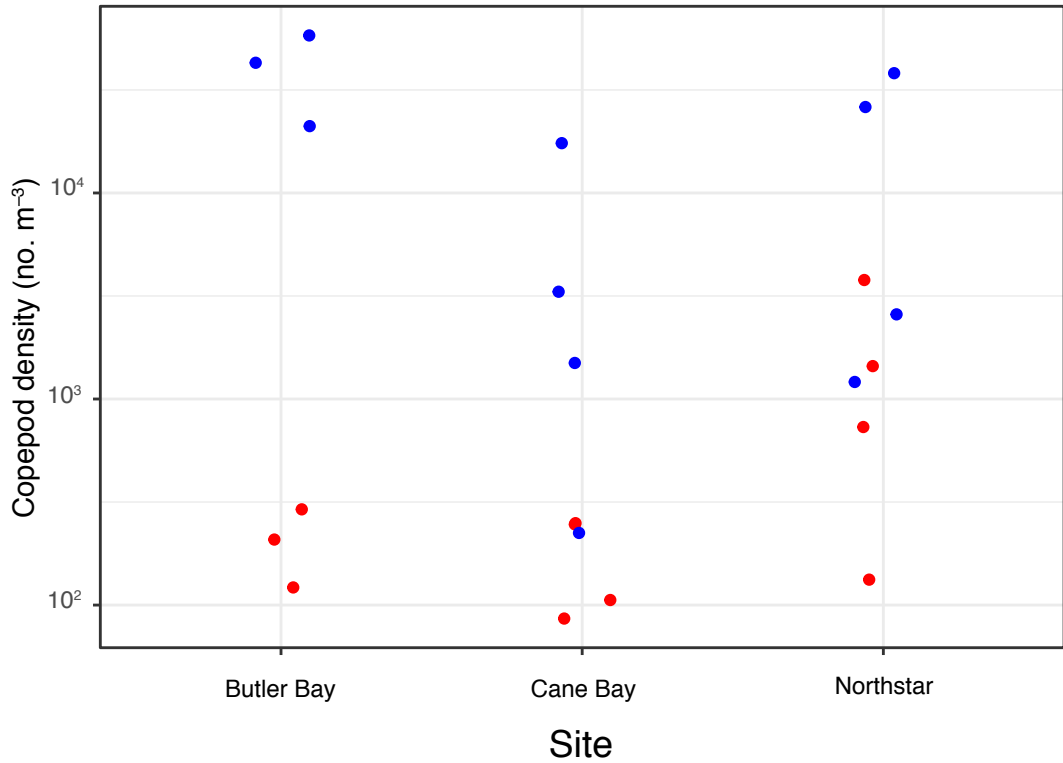

Supplement: Figure S4 — Each point is the mean copepod density (number of individuals/m3) on one sampling day in July 2012 (red symbols) or August 2012 (blue symbols). Points are jittered horizontally for visibility. [file peerj-08-8333-s004.pdf]

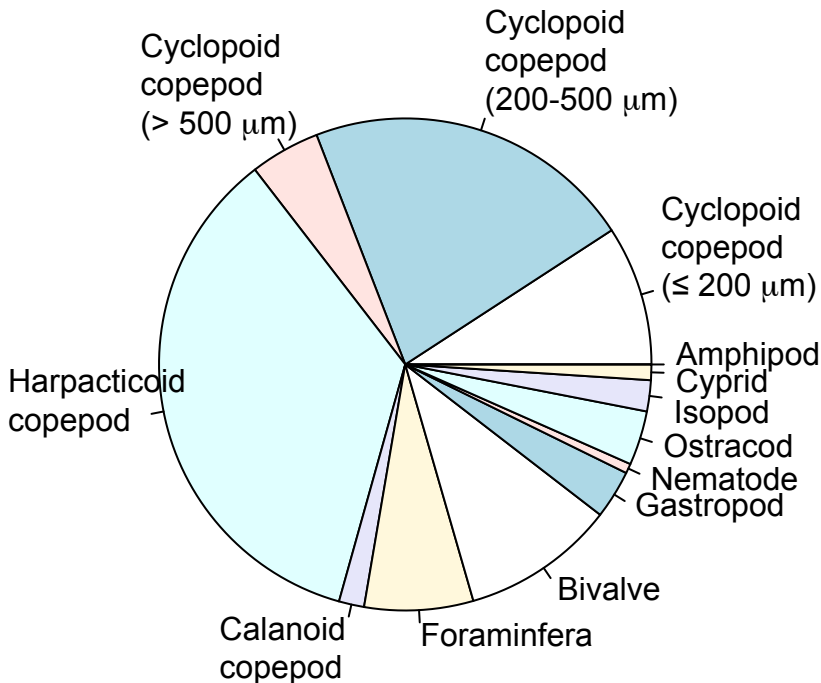

Supplement: Figure S5 — Diet items were identified to the lowest taxonomic level possible. The most common diet item, cyclopoid copepods, were also classified by carapace length. [file peerj-08-8333-s005.pdf]

(a)

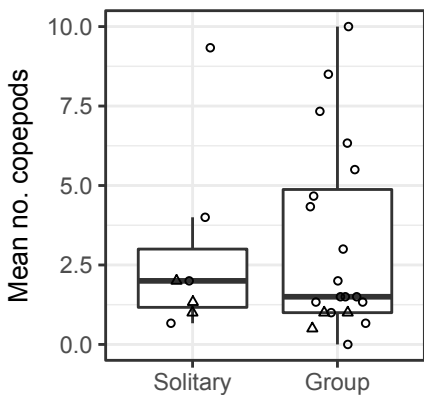

(b)

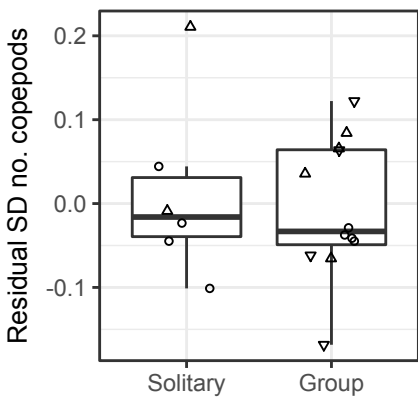

(c)

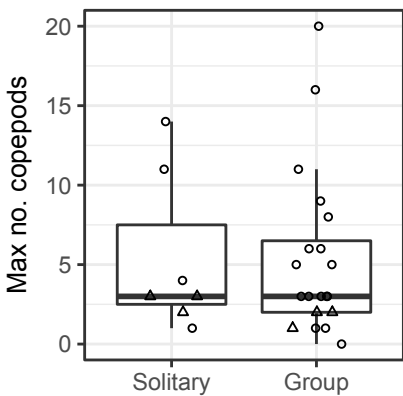

Supplement: Figure S6 — Relationships between different metrics of the number of copepods in guts of juvenile bluehead wrasse and group status (either solitary or grouped). The unit of replication is an individual group of fish or the sample of solitary fish on a particular reef and day. Each row shows a different diet statistic: (a)mean number of copepods in guts within a group; (b) standard deviation of number of copepods within a group; (c) maximum number of copepods within a group. Boxplots show median, interquartile range, and the 95% quantile range, along with individual data points. Data points are shown as triangles (data from July) or circles (data from August). [file peerj-08-8333-s006.pdf]

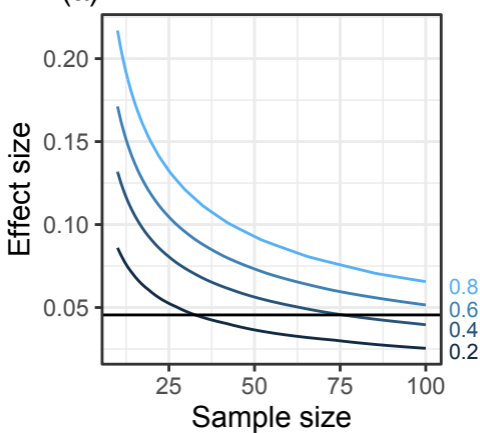

(b)

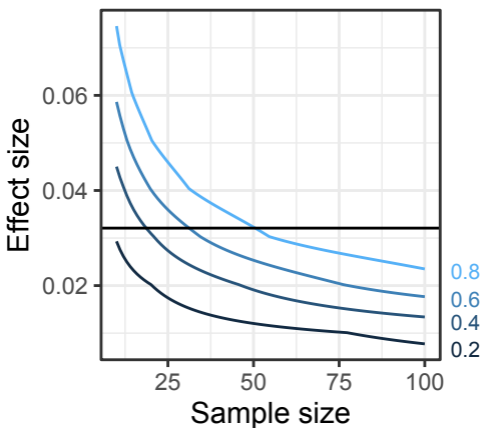

(c)

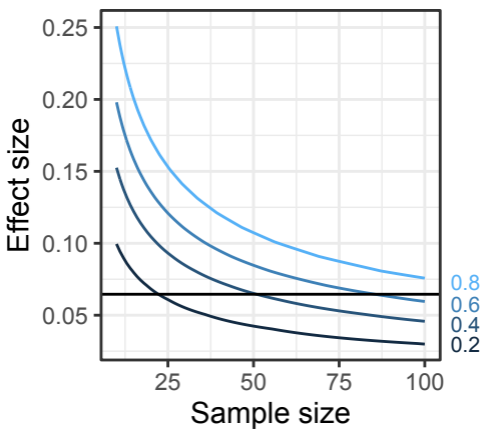

Supplement: Figure S7 — Panels show contour plots of power for the ’group size’ coefficient in linear models with the (a) mean, (b) standard deviation, or (c) maximum number of copepods in fish stomachs as the response variable, as function of effect size and sample size, given the observed sample variance. Colored curves are contours denoting specific levels of power (1-b). Each colored curve corresponds to the level of power indicated to the right by text of the same color. Black lines denote the observed sample size and effect size. [file peerj-08-8333-s007.pdf]

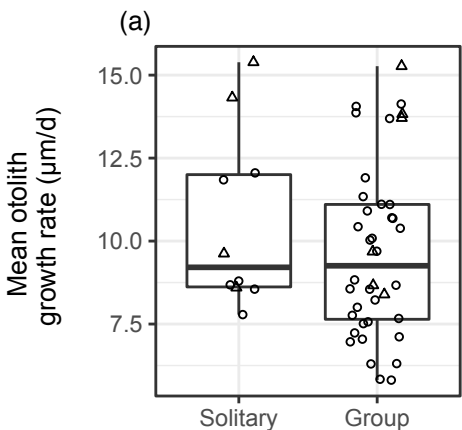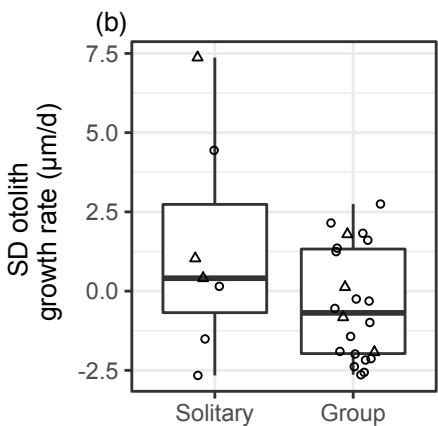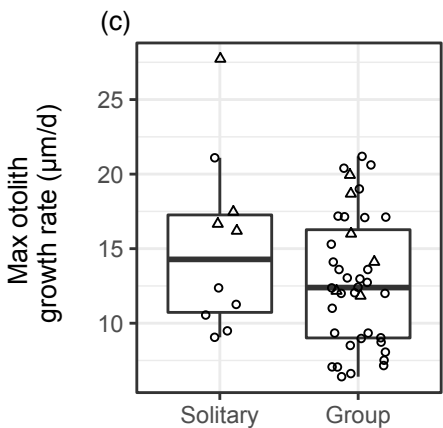

Supplement: Figure S8 — Relationships between different metrics of juvenile bluehead wrasse post-settlement growth rates (measured in otoliths) and group status (either solitary or grouped). The unit of replication is an individual group of fish or the sample of solitary fish on a particular reef and day. Each row shows a different diet statistic: (a) mean growth rate within a group; (b) standard deviation of growth rate within a group; (c) maximum growth rate within a group. Boxplots show median, interquartile range, and the 95% quantile range, along with individual data points. When there was a statistically meaningful effect of month, July and August points are shown as triangles and circles, respectively. [file peerj-08-8333-s008.pdf]

(a)

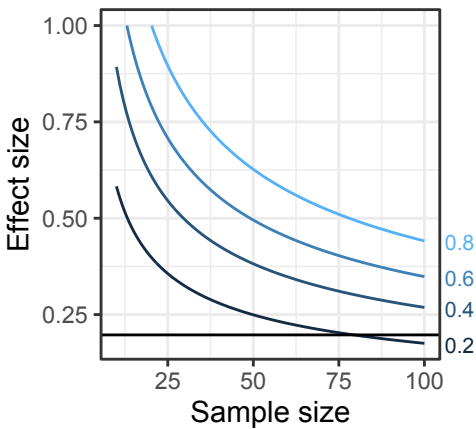

(b)

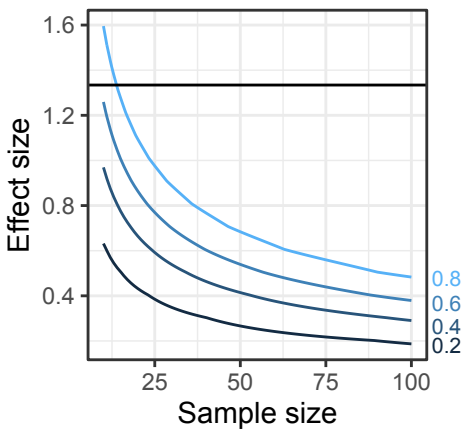

(c)

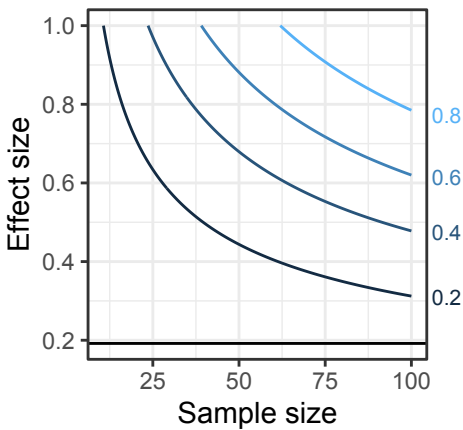

Supplement: Figure S9 — Panels show contour plots of power for the ’group size’ coefficient in linear models with the (a) mean, (b) standard deviation, or (c) maximum number of copepods in fish stomachs as the response variable, as function of effect size and sample size, given the observed sample variance. Colored curves are contours denoting specific levels of power (1b). Each colored curve corresponds to the level of power indicated to the right by text of the same color. Black lines denote the observed sample size and effect size. [file peerj-08-8333-s009.pdf]
