# Supplementary material for: Risk-sensitive foraging does not explain condition-dependent choices in settling reef fish larvae
Source: PeerJ. 2020 Jan 13;8:e8333. doi: 10.7717/peerj.8333 (PMC6964687; doi:10.7717/peerj.8333)
Supplement: Table S1 — Summary of the final models (after backwards elimination) for stomach contents. SE = standard error. There are two models for each response variable; one with a continous effect of group size and one with a binary effect of grouped vs. solitary. The response variables were log10(x + 1) transformed to meet the distribution assumptions of the tests. [file peerj-08-8333-s010.docx]

| **Term** | **Coefficient** | **SE** | ***t*** | ***p*** |
| --- | --- | --- | --- | --- |
| *Response: Mean* |  |  |  |  |
| Intercept | 0.29 | 0.16 | 1.87 | 0.07 |
| 1/[Group size] | 0.05 | 0.16 | 0.28 | 0.78 |
| Month (Aug) | 0.26 | 0.13 | 1.98 | 0.06 |
|  |  |  |  |  |
| Intercept | 0.35 | 0.13 | 2.76 | 0.01 |
| Group | –0.06 | 0.13 | -0.51 | 0.62 |
| Month (Aug) | 0.27 | 0.13 | 2.06 | 0.05 |
|  |  |  |  |  |
| *Response: SD* |  |  |  |  |
| Intercept | 0.38 | 0.042 | 9.10 | 5.3 x 10^–7^ |
| 1/[Group size] | 0.032 | 0.056 | 0.57 | 0.68 |
| log_10_(Plankton) | 3.7 x 10^-6^ | 1.5 x 10^-6^ | 2.46 | 0.029 |
| Site (Northstar) | –0.13 | 0.052 | –2.45 | 0.029 |
| Site (Butler Bay) | –0.15 | 0.087 | –1.73 | 0.11 |
|  |  |  |  |  |
| Intercept | 0.42 | 0.045 | 9.25 | 4.4 x 10^–7^ |
| Group | -0.033 | 0.045 | –0.73 | 0.48 |
| log_10_(Plankton) | 3.7 x 10^-6^ | 1.5 x 10^-6^ | 2.48 | 0.028 |
| Site (Northstar) | –0.13 | 0.051 | –2.49 | 0.027 |
| Site (Butler Bay) | –0.15 | 0.087 | –1.70 | 0.11 |
|  |  |  |  |  |
| *Response: Max* |  |  |  |  |
| Intercept | 0.44 | 0.18 | 2.48 | 0.021 |
| 1/[Group size] | 0.065 | 0.19 | 0.34 | 0.74 |
| Month (Aug) | 0.27 | 0.14 | 1.78 | 0.088 |
|  |  |  |  |  |
| Intercept | 0.54 | 0.15 | 3.67 | 0.001 |
| Group | –0.10 | 0.14 | –0.70 | 0.49 |
| Month (Aug) | 0.29 | 0.15 | 1.90 | 0.069 |
