# Supplementary material for: Risk-sensitive foraging does not explain condition-dependent choices in settling reef fish larvae
Source: PeerJ. 2020 Jan 13;8:e8333. doi: 10.7717/peerj.8333 (PMC6964687; doi:10.7717/peerj.8333)
Supplement: Table S2 — Summary of the final models (after backwards elimination) for otolith-derived growth. SE = standard error. There are two models for each response variable; one with a continous effect of group size and one with a binary effect of grouped vs. solitary. [file peerj-08-8333-s011.docx]

| **Term** | **Coefficient** | **SE** | ***t*** | ***p*** |
| --- | --- | --- | --- | --- |
| *Response: Mean* |  |  |  |  |
| Intercept | 11.86 | 1.00 | 11.85 | 1 x 10^–15^ |
| 1/[Group size] | -0.20 | 1.11 | -0.18 | 0.86 |
| Month (Aug) | -2.39 | 0.91 | –2.64 | 0.011 |
|  |  |  |  |  |
| Intercept | 11.93 | 0.95 | 12.53 | 1 x 10^–16^ |
| Group | -0.30 | 0.90 | -0.34 | 0.74 |
| Month (Aug) | -2.28 | 0.90 | –2.52 | 0.015 |
|  |  |  |  |  |
| *Response: SD* |  |  |  |  |
| Intercept | 6.39 | 1.61 | 3.98 | 6.0 x 10^-4^ |
| 1/[Group size] | 1.33 | 1.20 | 1.11 | 0.28 |
| log_10_(Plankton) | 8.46 x 10^-5^ | 4.52 x 10^–5^ | 1.87 | 0.074 |
| Site (Northstar) | -1.69 | 1.17 | –1.44 | 0.16 |
| Site (Butler Bay) | –3.54 | 1.89 | –1.87 | 0.07 |
| Month (Aug) | –3.25 | 1.46 | –2.22 | 0.036 |
|  |  |  |  |  |
| Intercept | 7.86 | 1.51 | 5.19 | 2.9 x 10^-5^ |
| Group | –1.33 | 0.99 | -1.34 | 0.19 |
| log_10_(Plankton) | 8.59 x 10^-5^ | 4.55 x 10^–5^ | 1.93 | 0.066 |
| Site (Northstar) | -1.67 | 1.16 | –1.44 | 0.16 |
| Site (Butler Bay) | –3.54 | 1.87 | –1.90 | 0.07 |
| Month (Aug) | –3.25 | 1.43 | –2.27 | 0.033 |
|  |  |  |  |  |
| *Response: Max* |  |  |  |  |
| Intercept | 17.19 | 1.78 | 9.65 | 9.8 x 10^-13^ |
| 1/[Group size] | –0.19 | 1.97 | –0.097 | 0.92 |
| Month (Aug) | –4.76 | 1.61 | –2.95 | 0.0050 |
|  |  |  |  |  |
| Intercept | 17.83 | 1.69 | 10.58 | 5.0 x 10^-14^ |
| Group | –1.25 | 1.60 | –0.78 | 0.44 |
| Month (Aug) | –4.40 | 1.60 | –2.75 | 0.008 |
